# Serum vitamin D is associated with improved lung function markers but not with prevalence of asthma, emphysema, and chronic bronchitis

**DOI:** 10.1038/s41598-020-67967-7

**Published:** 2020-07-09

**Authors:** Vijay Ganji, Asma Al-Obahi, Sumaya Yusuf, Zainab Dookhy, Zumin Shi

**Affiliations:** Human Nutrition Department, College of Health Sciences, QU Health, Qatar University, P.O.Box 2713, Doha, Qatar

**Keywords:** Physiology, Endocrinology

## Abstract

Hypovitaminosis D has been linked to several non-bone diseases. Relation between 25-hydroxyvitamin D [25(OH)D] and lung function and lung diseases has received little attention at the global level. Cross-sectional data from three National Health and Nutrition Examination Surveys, 2007–2008, 2009–2010, and 2011–2012 were used to investigate the relationship between serum 25(OH)D concentrations and lung function makers [forced vital capacity (FVC) and forced expiratory volume in 1 s (FEV1)] and lung diseases (asthma, emphysema, and chronic bronchitis) with multivariate regression models (n = 11,983; men, 6,010; women, 5,973). Serum 25(OH)D concentrations were directly associated with FVC and FEV1 (P for trend < 0.01). Individuals in the 4th quartile serum 25(OH)D had significantly higher FVC and FEV1 compared to those in the 1st quartile (P < 0.01). When data were stratified based on gender and smoking status, we found similar associations between serum 25(OH)D concentrations and lung function markers. There was no relation between serum 25(OH)D and prevalence of asthma, chronic bronchitis, and emphysema in US adults. Serum 25(OH)D concentration is associated with improved lung function markers but not with the prevalence of asthma, emphysema, and chronic bronchitis. Controlled studies are needed to determine if the vitamin D supplementation improves lung function in adults and in smokers.

## Introduction

Vitamin D, a lipophilic nutrient, is obtained from diet and supplements. It can also be synthesized in the skin from 7-dehydrocholesterol when exposed to Sun’s UV-B light. 25-dihydroxyvitamin D [25(OH)D] is the major circulatory form and a commonly used biomarker of vitamin D status^1^ although its use is questioned in studies linking 25(OH)D concentration with health outcomes^2^. The classical function of vitamin D is to maintain the calcium homeostasis. Recent evidence points a role for vitamin D in non-bone infirmities such as type-2 diabetes^3^, metabolic syndrome^4,5^, obesity^6^, cardiovascular diseases^7^, some cancers^8,9^, depression^10,11^, and infectious disease^12^. Vitamin D deficiency is a global health problem^13^. In the US, the vitamin D deficiency [< 50 nmol/L of 25(OH)D] is ~ 40% in 2010^14^. Vitamin D status is affected by skin pigmentation^15^, geographical location^16^, season^17^, body adiposity^18^, disease status^3–12^, exposure to sun light^19^, smoking^20^, race-ethnicity^21^, and use of sunblock loation^22^.

Asthma is a chronic inflammation of the airways resulting in wheezing sound and shortness of breath, cough, and chest pains. There is a growing incidence of asthma globally^23,24^. Previous studies on the relationship between vitamin D concentrations and asthma yielded equivocal results^25–28^. In patients with mild to moderate asthma, an inverse relationship was observed between vitamin D concentrations and number of asthma attacks^25–27^. A meta-analysis of studies from double-blind, randomized, placebo-controlled trials on adults (n = 658) showed that vitamin D reduced asthma exacerbation episodes^25^. Other studies suggested that vitamin D may be used for the treatment and control of asthma symptoms^26,27^. In contrast, a meta-analysis of seven studies found that vitamin D supplementation did not have an effect on reducing asthma severity or lung function^28^.

The relationship between serum vitamin D concentrations and lung function and lung diseases in the US national data has not been well investigated. A previous study on the US sample reported a direct relation between serum 25(OH)D concentrations and lung function markers and inverse association between 25(OH)D and prevalence of asthma^29^. In this current study, we combined three cycles of National Health and Nutrition Examination Survey (NHANES), 2007–2008, 2009–2010, and 2011–2012, into one working database. The relation between serum 25(OH)D and lung function in smokers is not well understood. Also, we studied the relation between serum 25(OH)D concentrations and other lung diseases such as emphysema, and chronic bronchitis in US adults. Therefore, the objective of this study was to investigate the relationship between serum 25(OH)D concentrations and lung function markers and lung diseases in US adults.

## Methods

### NHANES description

The National Center for Health Statistics (NCHS) conducts large, nationally representative sample surveys called NHANES on civilian US population, using a stratified, multistage, probability sample survey design. NHANESs were conducted as annual surveys since 1999. Data are released in 2-year cycles for public use. Data on demographic factors, food intake, and health were collected from personal interviews at the subjects’ homes. Physical examination and collection of blood and urine samples were performed in the Mobile Examination Center (MEC). Minorities such as low-income people, adolescents, ≥ 60 years old people, non-Hispanic blacks, and Mexican Americans/Hispanics were oversampled to produce reliable estimates for these groups. The description of the survey’s methodologies and analytic guidelines were reported in detail elsewhere^30^.

### Survey description

In this study, we used the data from NHANES 2007–2008, 2009–2010, and 2011–2012. These three surveys were combined into one analytic data file, NHANES 2007–2012. In short, NHANES 2007–2008 was conducted between January 2007 and December 2008 on 9,762 subjects. NHANES 2009–2010 was conducted from January 2009 to December 2010 on 10,253 subjects. NHANES 2011–2012 was conducted from January 2011 to December 2012 on 9,338 subjects.

### Study sample derivation

**Figure 1:**
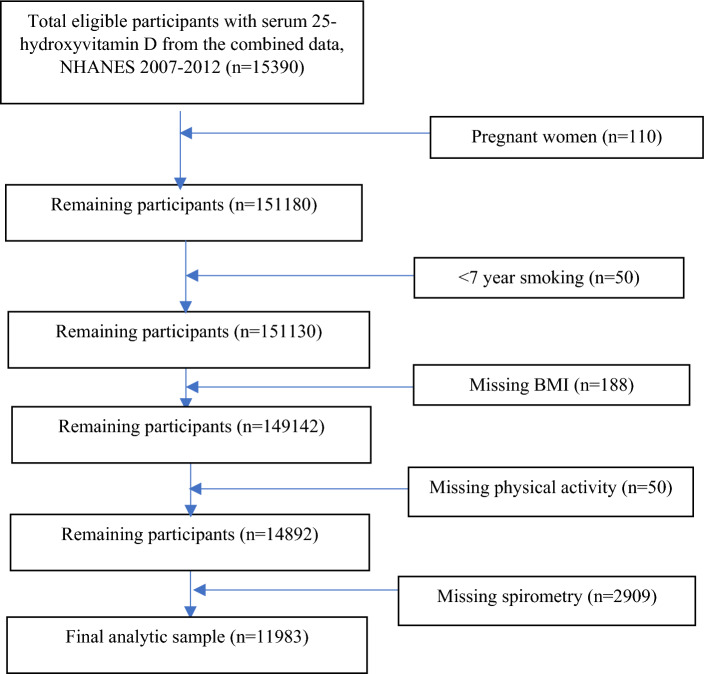
STROBE flow chart-sample selection criteria for the association between serum 25-hydroxyvitamin D concentration and lung function.

Data from ≥ 20 years and older participants from NHANES 2007–2012 were used in this study. Total participants with measured serum 25(OH)D concentration from the combined NHANES 2007–2012 cycles was 15,390. After the exclusion of pregnant women (n = 110) and subjects with missing data for smoking (n = 50), BMI (n = 188), physical activity (n = 50), and spirometry (n = 2,909), the final analytic sample was 11,983 subjects (weighted sample size, 163,363,012) (Fig. 1). The sample size for the analysis of relation between vitamin D and lung diseases such as asthma, emphysema, and chronic bronchitis were 11,972, 11,972, and 11,963, respectively.

### Vitamin D measurement

Liquid chromatography-tandem mass spectrometry (LC–TMS) was used to measure the serum 25(OH)D concentrations at the National Center of Environmental Health of CDC. LC–TMS is a better method because of improved analytical specificity and sensitivity compared to the methods that were used in the past. The analytical goals for imprecision and bias were ≤ 10% and ≤ 5%, respectively. Serum 25(OH)D concentration was defined as the sum of 25(OH)D_3_ and 25(OH)D_2_.

### Description of spirometry

Spirometry data were available only in NHANES 2007–2008, 2009–2010, and 2011–2012 cycles. Spirometry was performed as per the recommendations of the American Thoracic Society. Participants were not eligible for spirometry if they had painful infections, chest pain, physical problems with forceful expiration, history of an aneurysm or a detached retina, history of a collapsed lung or exposure to *Micobacterium tuberculosis*, heart disease, and hemoptysis. Subjects who were on supplemental oxygen were also not eligible for spirometry. Participants who had a history of congenital heart disease, hypertension, major arrythmia, or recently used the short-acting inhaled β2-agonist and participants who were pregnant or breastfeeding were excluded from spirometry. Two lung function markers were selected for the current analysis. These were forced vital capacity (FVC) and forced expiratory volume in 1 s (FEV1). Age-, gender-, and race-ethnicity-specific percent predicted values of FEV1 and FVC were calculated using the normal equations for spirometric parameters of the general US population.

### Description of confounding variables

Gender, age, race-ethnicity, BMI, poverty-income ratio (PIR), season of the survey, use of vitamin D supplements, alcohol intake, smoking, and sedentary activity were used as confounding variables. Age and BMI were used as continuous variables. Race-ethnicity was self- identified by the participants as non-Hispanic white, non-Hispanic black, Mexican American/Hispanic, and others. Smoking was defined as having blood cotinine concentrations > 1 ng/ml. PIR was calculated by dividing the total family income and the family’s poverty threshold. PIR was categorized into three groups, i.e., < 1.0 (low income), 1.0–2.5 (middle income), and ≥ 2.5 (higher income). Participants without any data for PIR were placed into the “not reported” category. Vitamin D supplement use was identified by the participant’s answer to the question “Did you take supplements in the past 30 days?”. Information about alcohol drinking was collected by asking “Have you had at least 12 alcohol drinks/one year?” The responses were divided into three categories: yes, no, and not reported. Sedentary activity is the time usually spent on sitting or reclining (at work, at home, or at school) on a typical day. This also included time spent sitting at a desk, sitting with friends, traveling in a car, bus, or train, reading, watching television, or using a computer. This did not include time spent sleeping.

### Description of asthma, chronic bronchitis, and emphysema

The medical condition of the participants was self-reported by answering several questions during the personal interview. Participants who answered “yes” to the following five questions were considered having asthma: “Has a doctor or other health professional ever told you that you have asthma?”, “Do you still have asthma?”, “During the past 12 months, have you had an episode of asthma or an asthma attack?”, “During the past 12 months, have you had to visit an emergency room or urgent care center because of asthma?”, and “During the past three months, have you taken medication prescribed by a doctor or other health professionals for asthma?”. For chronic bronchitis, the participants who answered “yes” to the following two questions were considered having chronic bronchitis “Has a doctor or other health professional ever told you that you had chronic bronchitis?” and “Do you still have chronic bronchitis?”. Regarding the emphysema, the participants who answered “yes” to the following question were considered as having emphysema “Has a doctor or other health professional ever told you that you had emphysema?”.

### Statistical analysis

Data analysis was performed with STATA software (STATA, Version 16, College Station, TX, USA). To produce statistically reliable estimates, a 6-year sample weights were used taking the complex survey design into account as per NCHS guidelines. The variance was estimated with the Taylor Linearization method. The sample weights and variance estimation methods were described in detail in the NHANES Analytic Guidelines^30^. Serum 25(OH)D concentrations were stratified into quartiles. The proportion of subjects with lung function diseases (asthma, emphysema, and bronchitis) across the quartiles of serum 25(OH)D was compared using the χ^2^ test. The association between prevalence of lung function diseases and serum 25(OH)D concentration was analyzed with univariate and multivariable logistic regression analysis. Multivariable-adjusted odds ratios (OR) and 95% CI were derived for the presence of lung function diseases for each serum 25(OH)D concentration quartile category after adjusting the analysis for gender, age, race-ethnicity, sedentary activity, PIR, serum cotinine (smoking), alcohol intake, vitamin D supplements intake, season of examination, and BMI. ORs for the likelihood of having lung function disease across the quartiles of serum 25(OH)D concentration were compared with Bonferroni correction for multiple comparison.

Unadjusted and adjusted means for FVC and FEV1 across the serum 25(OH)D quartiles were generated using multivariable regression models. Multivariate models were adjusted for gender, age, race-ethnicity, sedentary activity, PIR, serum cotinine, alcohol intake, vitamin D supplements intake, season of examination, and BMI. Interactions between serum 25(OH)D and confounding variables were determined and these interaction terms were included in the analysis. Because interactions between serum 25(OH)D concentrations and sex and smoking status were significant, we performed a separate subgroup analysis for the association between serum 25(OH)D and FVC and FEV1 by smoking status and gender. Pairwise comparisons between the means of FVC and FEV1 across the four quartiles of serum 25(OH)D were performed with Bonferroni correction after testing the hypothesis with Wald F-test. A P-value of ≤ 0.05 was considered statistically significant in all analyses.

### Ethical approval and consent to participate

All NHANES study protocols were approved by the Ethics Review Committee of NCHS of CDC and all subjects written consented before participation. The reasech was conducted in accordance with all the relevant guidelines and regulations.

## Results

### Subject characteristics of the study population

The characteristics of study participants are shown in Table 1. From the combined three cycles of NHANES 2007–2012, a total of 11,983 participants (men, 6,010; women, 5,973) ≥ 20 years old, who had serum 25(OH)D concentrations measured were included in the current analysis. Gender, age, race-ethnicity, smoking status, alcohol intake, season of examination, BMI, vitamin D supplement intake, sedentary activity, and PIR were significantly related to the serum 25(OH)D concentrations. It was observed that the higher the age, the higher the serum 25(OH)D concentrations (P < 0.001). Subjects with high serum 25(OH)D were more likely to be non-Hispanic white (70%) compared to non-Hispanic black (9%) and Mexican American/Hispanic (7%). Among smokers, the highest proportion of subjects were in the lowest serum 25(OH)D quartile category (37%). On the other hand, among non-smokers, the highest proportion of subjects were in the highest 25(OH)D quartile category (73%). Individuals with low BMI had higher serum 25(OH)D than those with high BMI. Among non-supplement users, a higher proportion of subjects were in the lowest serum 25(OH)D quartile (86%) whereas, among supplement users, a higher proportion of subjects were in the highest serum 25(OHD) quartile category.

**Table 1:** Subject characteristics of study population (n = 11,983): National Health and Nutrition Examination Surveys 2007–2012 [mean ± SD or *n* (%)].

		Serum 25(OH)D quartiles			*P*-value
	Q1 (*n* = 2,995)	Q2 (*n* = 2,996)	Q3 (*n* = 2,996)	Q4 (*n* = 2,996)	
Serum 25(OH)D, nmol/L	32 ± 8	53 ± 5	69 ± 5	97 ± 19	< 0.001
Age, years	44 ± 16	45 ± 16	48 ± 16	51 ± 16	< 0.001
**Sex**					< 0.001
Men, *n*	1,410 (47%)	1646 (55%)	1625 (54%)	1,329 (44%)	
Women, *n*	1585 (53%)	1,350 (45%)	1,371 (46%)	1667 (56%)	
**Race-ethnicity**					< 0.001
Non-Hispanic white, *n*	541 (18%)	1,022 (34%)	1563 (52%)	2099 (70%)	
N-Hispanic black, *n*	1,250 (42%)	600 (20%)	377 (13%)	271 (9%)	
Mexican American/Hispanic, *n*	579 (19%)	662 (22%)	455 (15%)	202 (7%)	
Others, *n*	625 (21%)	712 (24%)	601 (20%)	424 (14%)	
**Smoking status**					< 0.001
Non-smoker, *n*	1903 (64%)	2089 (70%)	2,146 (72%)	2,194 (73%)	
Smoker, *n*	1,092 (37%)	907 (30%)	850 (28%)	802 (27%)	
**Alcohol consumption**					< 0.001
No, *n*	455 (15%)	490 (16%)	482 (16%)	440 (15%)	
Yes, *n*	1897 (63%)	1923 (64%)	2028 (68%)	2091 (70%)	
Not reported, *n*	643 (22%)	583 (20%)	486 (16%)	465 (15%)	
**Season of examination**					< 0.001
Winter, *n*	1826 (61%)	1507 (50%)	1,232 (41%)	964 (32%)	
Summer, *n*	1,169 (39%)	1,489 (50%)	1764 (59%)	2032 (68%)	
**BMI, kg/m**	31 ± 8	30 ± 7	29 ± 6	27 ± 6	< 0.001
**Supplement use**					< 0.001
No, *n*	2,579 (86%)	2,236 (75%)	1815 (61%)	1,275 (43%)	
Yes, *n*	416 (14%)	760 (25%)	1,181 (39%)	1721 (57%)	
**Sedentary activity**					< 0.001
< 3 h, *n*	971 (32%)	1,093 (37%)	1,029 (34%)	963 (32%)	
3–6 h, *n*	971 (32%)	992 (33%)	1,054 (35%)	1,130 (38%)	
> 6 h, *n*	1,053 (35%)	911 (30%)	913 (31%)	903 (30%)	
**Poverty income ratio**					< 0.001
< 1.30, *n*	1,024 (37%)	943 (35%)	818 (30%)	704 (25%)	
1.3–3.5, *n*	1,068 (39%)	1,019 (37%)	982 (36%)	930 (33%)	
> 3.5, *n*	650 (24%)	767 (28%)	960 (35%)	1,166 (42%)	

### Serum 25(OH)D and markers of lung function

The association between serum 25(OH)D concentrations and the markers of lung function in US adults is described in Table 2. In the multivariate-adjusted models, serum 25(OH)D concentrations were significantly, directly associated with lung function markers, i.e., FVC and FEV1 (P for trend < 0.001). Furthermore, individuals in the 4th quartile serum 25(OH)D had significantly higher FVC and FEV1 compared to those in the 1st quartile serum 25(OH)D concentration (P < 0.001).

**Table 2:** Association between serum 25(OH)D concentrations and markers of lung function in US adults (n = 11,983): National Health and Nutrition Examination Surveys 2007–2012 (mean ± SE).

	Serum 25(OH)D quartiles				*P*-value
	Q1 (*n* = 2,995 )	Q2 (*n* = 2,996)	Q3 (*n* = 2,996)	Q4 (*n* = 2,996)	
**FVC, mL**					
Unadjusted	3,000 ± 20^a^	3,300 ± 120^b^	3,310 ± 30^b^	3,180 ± 30^b^	0.005
Multivariate-adjusted	3,110 ± 20^a^	3,200 ± 180^b^	3,240 ± 20^b, c^	3,260 ± 20^c^	< 0.001
**FEV1, mL**					
Unadjusted	3,800 ± 30^a^	4,180 ± 130^b^	4,260 ± 30^b^	4,150 ± 30^c^	< 0.001
Multivariate-adjusted	4,010 ± 20^a^	4,090 ± 190^b^	4,140 ± 20^b^	4,190 ± 20^b^	< 0.001

Means not sharing common superscripts are significantly different from each other within the row. Multivatiate analysis was adjusted for gender, age, race-ethnicity, sedentary activity, PIR, smoking status, alcohol intake, vitamin D supplements intake, season of examination, and BMI.

Association between serum 25(OH)D concentrations and FVC and FEV1 by smoking status and sex in US adults is presented in Table 3. Similarly, in this subgroup analysis, serum 25(OH)D concentrations were significantly, directly associated with the markers of lung function in both smokers and non-smokers and in both men and women.

**Table 3:** Association between serum 25(OH)D concentrations and markers of lung function by sex and smoking status in US adults: National Health and Nutrition Examination Surveys 2007–2012 (mean ± SE).

	Serum 25(OH)D quartiles						*P*-value
	Q1 (*n* = 2,995)	Q2 (*n* = 2,996)		Q3 (*n* = 2,996)	Q4 (*n* = 2,996)		
**FVC, mL**							
Smoking status							0.04
Non-smoker	4,010 ± 20^a^	4,080 ± 170^b^		4,140 ± 20^b,c^	4,210 ± 20^c^		
Smoker	4,050 ± 30^a^	4,140 ± 120^a,b^		4,190 ± 40^b^	4,150 ± 30^a,b^		
Gender							< 0.001
Men	4,620 ± 40^a^	4,770 ± 140^a,b^		4,850 ± 30^b,c^	4,960 ± 30^c^		
Women	3,430 ± 20^a^	3,460 ± 170^a,b^		3,460 ± 20^a,b^	3,460 ± 20^b^		
**FEV1, mL**^**e**^							
Smoking status							0.002
Non-smoker	3,130 ± 20^a^	3,210 ± 150^b^		3,280 ± 10^b,c^	3,320 ± 20^c,d^		
Smoker	3,080 ± 30^a^	3,190 ± 110^b^		3,170 ± 30^a,b^	3,130 ± 30^a,b^		
Gender							0.009
Men	3,550 ± 30^a^	3,700 ± 120^b^		3,760 ± 20^b^	3,800 ± 30^b^		
Women	2,690 ± 20^a^	2,720 ± 140^a,b^		2,740 ± 20^a,b^	2,750 ± 20^b,c^		

Means not sharing common superscripts are significantly different from each other within the row. Analysis was adjusted for gender (only for smoking status model), age, race-ethnicity, sedentary activity, PIR, smoking (only for gender model), alcohol intake, vitamin D supplements intake, season of examination, and BMI. P-values are significance for interactions between serum 25(OH)D and smoking status or gender.

### Serum 25(OH)D concentrations and asthma, emphysema, and chronic bronchitis

The association between serum 25(OH)D and prevalence of asthma, emphysema, and chronic bronchitis in US adults is described in Table 4. Across the serum 25(OH)D concentration quartiles, the prevalence of asthma, emphysema, and chronic bronchitis ranged from 13% to 16%, 0.9% to 1.7%, and 4.5% to 5.4%, respectively. No significant relationship was observed between serum 25(OH)D concentrations and the prevalence of asthma, emphysema, and chronic bronchitis in both unadjusted and multivariate-adjusted analysis.

**Table 4:** Relationship between serum 25(OH)D concentrations and prevalence of asthma, emphysema, and chronic bronchitis in US adults: National Health and Nutrition Examination Surveys 2007–2012 [odds ratio (95% confidence intervals)].

	Serum 25(OH)D quartiles				*P*-value
	Q1	Q2	Q3	Q4	
**Asthma**					
*n* Cases	2,994 446	2,994 399	2,991 385	2,993 403	
Prevalence, %	16.0	14.6	13.2	13.4	0.18
Unadjusted OR (95% CI)	1.23 (0.98–1.55)	1.10 (0.86–1.42)	0.98 (0.79–1.22)	1.0	0.09
Multivariable-adjusted OR (95% CI)	1.19 (0.95–1.51)	1.13 (0.87–1.46)	1.01 (0.80–1.28)	1.0	0.14
**Emphysema**					
*n* Cases	2,993 39	2,993 29	2,991 34	2,995 54	
Prevalence, %	1.7	0.9	1.3	1.4	0.19
Unadjusted OR (95% CI)	1.19 (0.76–1.88)	0.62 (0.36–1.06)	0.95 (0.58–1.54)	1.0	0.99
Multivariable-adjusted OR (95% CI)	1.11 (0.66–1.87)	0.69 (0.36–1.31)	0.60 (0.36–0.99)	1.0	0.89
**Chronic bronchitis**					
*N*	2,993	2,991	2,989	2,990	
Cases	147	115	142	176	
Prevalence, %	5.4	4.5	4.9	5.4	0.58
Unadjusted OR (95% CI)	0.99 (0.71–1.37)	0.82 (0.57–1.17)	0.89 (0.61–1.31)	1.0	0.67
Multivariable-adjusted OR (95% CI)	1.06 (0.71–1.57)	0.98 (0.64–1.49)	0.95 (0.65–1.39)	1.0	0.84

## Discussion

In this study, we investigated the relationship between serum 25(OH)D concentrations and lung function markers and a few selected lung diseases such as asthma, emphysema, and chronic bronchitis. We found that serum 25(OH)D concentrations are directly associated with lung function markers. This is the first study to investigate the association between 25(OH)D concentrations and lung function markers and lung diseases (asthma, emphysema, and chronic bronchitis) on the US population using the nationally representative data from three cycles of NHANES 2007–2012. Also, we are first to report that in both smokers and non-smokers, vitamin D concentrations are directly associated with lung function markers in US adults.

In this study, serum 25(OH)D concentrations are directly associated with FCV and FEV1. Also in smokers, we observed a similar relationship with lung function markers. Similar to our findings, a meta-analysis on 27 studies suggested that serum 25(OH)D concentration was directly associated with lung function in asthma patients^31,32^. Another study conducted on 2,607 adolescents aged 15 years found that serum 25(OH)D concentrations were directly associated with FVC and FEV_1_. This association was stronger for FVC than FEV_1_^33^. Also in COPD patients, a similar association was found^34^.

In contrast to our findings, other studies found a negative association between vitamin D concentrations and lung function. In a randomized controlled trial on 442 adults (aged 50–84 years old), the subjects received a high-dose of vitamin D supplementation on a monthly basis for 1.1 years. There was no significant improvement in lung function although serum 25(OH)D concentrations were improved to > 50 nmol/L. In a subgroup analysis, they found that lung function markers (FEV1 and FEV z-score) were improved with vitamin D supplementation in ever smokers, in particular those with vitamin D deficiency^35^. A cross-sectional study with 1551 men and 1,391 women did not confirm a positive association between serum 25(OH)D concentrations and lung function after controlling for several confounding variables. In COPD patients, a weak relationship was observed between serum vitamin D and FEV1/FVC^36^. This is more likely due to less sample size^37^.

Vitamin D has an anti-inflammatory effect that can be modified by exposure to cigarette smoke^38^. Exposure to cigarette smoking and environmental tobacco smoke were associated with decreased 25(OH)D concentration in the blood^39^. In a longitudinal cohort study on white men (≥ 20 years old), vitamin D deficiency in relation to lung function was examined. In this cohort, no association was found between vitamin D deficiency and lung function. In a meta-analysis study, vitamin D did not improve lung function decline in subjects with chronic obstructive pulmonary disease^40^ .Similar to our results, a study conducted on Chinese found that current smokers had lower vitamin D compared to never smokers, and that relation showed a dose–response pattern. Longer duration after quitting smoking was correlated to a higher concentration of (25OH)D than the current smokers (P = 0.04)^41^. This suggests that vitamin D may have a protective effect on lungs against the damage caused by smoking, due to its anti-inflammatory properties^38^. In the same cohort, investigators found that vitamin D induces the production of antioxidants. This may be beneficial to counteract the effects of oxidative stress caused by cigarette smoking. It was found that cigarette smoke decreases the production of the active form of vitamin D and affects the expression of vitamin D receptors. Additionally, subjects with vitamin D deficiency with many years of smoking had greater lung function decline^38^.

In our study, the association between serum 25(OH)D and the prevalence of asthma, emphysema, and chronic bronchitis was not statistically significant in both unadjusted and multivariate-adjusted models. However, there was a trend toward decrease in the prevalence of asthma as the serum 25(OH)D concentrations increase. Studies relating vitamin D with asthma has yielded equivocal results. A cross-sectional study based on NHANES data found no association between low concentration of serum 25(OH)D and asthma^42^. Further, a recent study reported no effect of vitamin D supplementation in pregnant women on the prevalence of asthma and recurrent wheeze in their offspring^43^. A randomized control trial that was done in Saudi adults (n = 1,070) found that the association between 25(OH)D concentration and self-reported asthma was not statistically significant in age, gender, and BMI adjusted model. The study also found that the percentage of participants with vitamin D deficiency was slightly higher in people with asthma compared to the control group^44^. On the other hand, a study found an association between serum 25(OH)D and incidence of asthma. The risk of asthma was significantly increased with each 25 nmol/L reduction of serum 25(OH)D^45^. Some studies showed a relationship between serum 25(OH)D concentration and asthmatic exacerbation. Vitamin D deficient patients tend to have an increase in the frequency of asthma exacerbation and vitamin D supplementation was shown to be effective in reducing asthma exacerbation^27,46,47^. Limited evidence from randomized controlled studies suggested that in current and former smokers supplementation of vitamin D improved lung function and reduced the risk of asthma^48^.The discrepancy between our study and other studies may be due to sample size and the confounding variables used in the multivariate adjusted analysis. Although serum 25(OH)D concentrations are associated with improved lung function markers, it may not have stronger effect to make an impact on the lung disease prevalence.

Due to the cross-sectional nature of this study, the cause and effect relationship is not possible. The disease diagnosis was self-reported by participants, so the diagnosis of lung diseases may have been under reported. Because this study was based on nationally representative sample survey, results can be applied to US population at large. In conclusion, serum 25(OH)D was directly associated with lung function markers (FVC and FEV1) in US adults. Also, in a separate analysis, a similar association was confirmed between serum 25(OH)D concentration and lung function markers in smokers and non-smokers and in men and women. However, serum 25(OH)D was not significantly associated with the prevalence of asthma, emphysema, and chronic bronchitis. Adequately powered, randomized controlled studies are needed to examine if vitamin D supplementation would improve lung function markers in general population and specifically in smokers with compromised lung function.

## Data Availability

All the data supporting the findings within the article and its supplementary files will be available from the corresponding author upon request (V.G).

## Abbreviations

25(OH)D: 25-Hydroxyvitamin D
BMI: Body mass index
FVC: Forced vital capacity
FEV1: Forced expiratory volume in 1 s
LC-TMS: Liquid chromatography-tandem mass spectrometry
NCHS: National Center for Health Statistics
NHANES: National Health and Nutrition Examination Survey
OR: Odds ratio
PIR: Poverty income ratio
