# Author Correction: Serum vitamin D is associated with improved lung function markers but not with prevalence of asthma, emphysema, and chronic bronchitis

**DOI:** 10.1038/s41598-020-70344-z

**Published:** 2020-08-19

**Authors:** Vijay Ganji, Asma Al-Obahi, Sumaya Yusuf, Zainab Dookhy, Zumin Shi

**Affiliations:** Human Nutrition Department, College of Health Sciences, QU Health, Qatar University, P.O.Box 2713, Doha, Qatar

Correction to:* Scientific Reports* 10.1038/s41598-020-67967-7, published online 09 July 2020

The original version of this Article contained a typographical error in the spelling of the author Zainab Dookhy, which was incorrectly given as Zinab Dookhy. This has now been corrected in the PDF and HTML versions of the Article.

